# Revolutionising dental technologies: a qualitative study on dental technicians’ perceptions of Artificial intelligence integration

**DOI:** 10.1186/s12903-023-03389-x

**Published:** 2023-09-25

**Authors:** Galvin Sim Siang Lin, Yook Shiang Ng, Nik Rozainah Nik Abdul Ghani, Kah Hoay Chua

**Affiliations:** 1 Department of Dental Materials, Faculty of Dentistry, Asian Institute of Medicine, Science and Technology (AIMST) University, 08100 Bedong, Kedah Malaysia; 2 Conservative Dentistry Unit, School of Dental Sciences, Universiti Sains Malaysia, Health Campus, 16150 Kubang Kerian, Kelantan Malaysia; 3 Department of Dental Technology, Faculty of Dentistry, Asian Institute of Medicine, Science and Technology (AIMST) University, 08100 Bedong, Kedah Malaysia

**Keywords:** Artificial intelligence, Computer-aided design, Dental technician, Dental technology, Qualitative study

## Abstract

**Background:**

The integration of artificial intelligence (AI) in dentistry has the potential to revolutionise the field of dental technologies. However, dental technicians’ views on the use of AI in dental technology are still sparse in the literature. This qualitative study aimed to explore the perceptions of dental technicians regarding the use of AI in their dental laboratory practice.

**Methods:**

Twelve dental technicians with at least five years of professional experience and currently working in Malaysia agreed to participate in the one-to-one in-depth online interviews. Interviews were recorded, transcribed verbatim and translated. Thematic analysis was conducted to identify patterns, themes, and categories within the interview transcripts.

**Results:**

The analysis revealed two key themes: “Perceived Benefits of AI” and “Concerns and Challenges”. Dental technicians recognised the enhanced efficiency, productivity, accuracy, and precision that AI can bring to dental laboratories. They also acknowledged the streamlined workflow and improved communication facilitated by AI systems. However, concerns were raised regarding job security, professional identity, ethical considerations, and the need for adequate training and support.

**Conclusion:**

This research sheds light on the potential benefits and challenges associated with the integration of AI in dental laboratory practices. Understanding these perceptions and addressing the challenges can support the effective integration of AI in dental laboratories and contribute to the growing body of literature on AI in healthcare.

## Background

The field of dental technology encompasses various tasks involved in the fabrication of dental prostheses, such as veneers, crowns, bridges, and dentures, in order to restore oral function and aesthetics [1, 2]. Traditionally, dental technicians have relied on manual techniques and specialised craftsmanship to create these prostheses [3]. Nonetheless, the field of dental technology has undergone significant transformations in recent years due to advancements in technology. One such advancement that has gained considerable attention is the integration of artificial intelligence (AI) in dental practice [4]. AI refers to the ability of machines to simulate human intelligence, enabling them to perform tasks that typically require human cognition [5].

Undeniably, advancements in digital dentistry and AI present new opportunities to optimise and automate certain aspects of the dental laboratory workflow [6]. In the context of dental technology, AI can support dental technicians in various ways, including digital impressions, computer-aided design / computer-aided manufacturing (CAD/CAM), image analysis, and quality control [1, 6]. Moreover, AI algorithms can analyse vast amounts of dental data, recognise patterns, and generate predictions or recommendations to aid dental technicians in their decision-making processes [7]. For instance, AI algorithms can analyse digital impressions [8], generate customised designs [9], and provide instructions for automated milling or three-dimensional printing of prosthetic restorations [10]. This not only improves the fit and aesthetics of dental prostheses, but also reduces manual labour and turnaround time in dental laboratories [11, 12]. It is worth noting that AI has the potential to revolutionise the way dental technicians work, offering opportunities for increased efficiency, accuracy, and productivity in dental laboratories. However, the successful implementation of AI in dental technology relies not only on its technical capabilities but also on the perceptions and acceptance of dental professionals, particularly dental technicians.

Dental technicians play a pivotal role in the fabrication process of various dental prostheses [13], and their acceptance and engagement with AI technologies are crucial for the implementation’s effectiveness. Understanding the perceptions of dental technicians is pertinent for effectively incorporating AI into dental technology and ensuring its successful adoption within dental laboratories. While there is existing research on AI applications in dentistry [7, 14, 15], information on dental technicians’ perspectives, specifically on the use of AI, is still sparse in the literature. Hence, this qualitative study aims to explore the perceptions of dental technicians regarding the use of AI in the field of dental technology. By delving into their concerns, considerations and expectations, the current research can shed light on the potential benefits and challenges associated with the integration of AI in dental laboratory practices. Furthermore, this research will also contribute to the growing body of literature on the integration of AI in healthcare.

## Methods

### Ethical considerations

Ethical approval was obtained from the first author (GSSL) institutional review board (ethical approval code: AUHEC/FOD/2023/02/07/11) prior to data collection. Participants were provided with informed consent forms and assured of the confidentiality and anonymity of their responses.

### Study design

The present exploratory qualitative study adopted a one-to-one in-depth interview approach to gather rich and detailed data about the perceptions of dental technicians regarding the use of AI in dental technology. The use of one-to-one interview allowed for an in-depth exploration of participants’ experiences, beliefs, and attitudes [16]. Furthermore, the Consolidated Criteria for Reporting Qualitative Research (COREQ) checklist was used to report the current study data [17] (Appendix 1).

### Theoretical framework

The Technology Acceptance Model (TAM) was used as the theoretical framework of the present study. It is a widely used theoretical framework for understanding users’ acceptance and adoption of technology [18]. It focuses on perceived usefulness and perceived ease of use as key determinants of users’ attitudes and intentions towards using a particular technology. In the current study, TAM was applied to examine dental technicians’ perceptions of AI in dental technology, specifically in terms of their perceived benefits and concerns.

### Research team and participants

The interview was conducted by the first investigator (GSSL) who is currently a male dental lecturer with a dental materials science postgraduate qualification (at the time the study was conducted). The interviewer had extensive experience in conducting qualitative study with special interest in dental education. Twelve dental technicians with at least five years of professional experience and currently working in Malaysia were selected using a purposive sampling via e-mail, WhatsApp, phone call and face-to-face. All twelve dental technicians agreed to participate in the study and gave their consent. The participants were recruited from different dental laboratories within the nation, ensuring a diverse range of experiences and perspectives. Moreover, the study participants had no direct relationship with the interviewer to avoid bias. They were also informed that the aim of the present was to explore dental technicians’ perceptions of the use of AI in dental technology.

### Data collection

The interview was conducted between March to April 2023, using an online Zoom platform with no other individual was present during the interviews apart from the interviewer and respective participant. Semi-structured interviews were conducted with each participant, employing a pre-designed and pre-piloted interview guide. The guide was prepared in both English and Malay versions. It consisted of open-ended questions and prompts to encourage participants to discuss their perceptions of AI in dental technology, its potential benefits, challenges, and concerns. Interviews lasted between 20 and 35 min and were audio-recorded with participants’ consent and later transcribed verbatim for analysis using NVivo software 12.6 (QSR International Pty Ltd). In the present study, we employed a clean verbatim, whereby nonverbal sounds, repetitions, and filler words like *“uh”*, *“um”*, and *“ya”* were omitted. Since some of the dental technicians responded in Malay language, a qualified translator who is fluent in both Malay and English languages was invited to translate the transcript into English language. No repeat interview was carried out and data saturation was achieved after interviewing the 10th participant. However, the interview sessions were conducted with the 11th and 12th participants to allow for a richness of information.

### Data analysis

Thematic analysis was employed to identify patterns, themes, and categories within the interview transcripts. The analysis involved several iterative stages, including familiarisation with the data, coding, theme development, and interpretation. The analysis process was conducted individually by two investigators (GSSL, KHC), ensuring a rigorous and systematic approach. Any coding disputes were addressed, resolved, and corrected as necessary through discussion among the two investigators. Participants were also asked to proofread the results and they agreed that no new categories or themes are found. Exemplary quotations that have been pseudonymised were given to each participant to ensure participant’s privacy [19]. Pseudonymisation number P stands for participant. In addition, the themes that emerged in the current study were aligned to form an explicatory model in a Venn diagram [20].

## Results

**Fig. 1:**
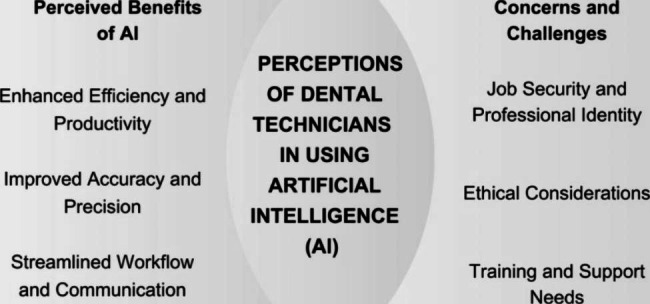
Venn diagram showing the perceptions of dental technicians of using AI in dental technology

Six participants in the present study had 5 to 10 years of experience as dental technicians with a basic diploma qualification in dental technology. Meanwhile, the remaining five participants had 11 to 15 years of experience, with one participant had more than 15 years of working experience. The sample included individuals from diverse backgrounds, working in different types of dental laboratories, including public sectors, private practices, and academic institutions as listed in Table 1. Thematic analysis revealed two key themes (“Perceived benefits of AI” and “Concerns and challenges”) and several subthemes that encompassed the participants’ perceptions of AI in dental technology (Fig. 1).

**Table 1:** Dental technicians’ demographic background

Items	respondent (*n*)
Gender	
Male	9
Female	3
*Total*	12
Years of experience	
5 to 10 years	6
11 to 15 years	5
More than 15 years	1
*Total*	12
Affiliation	
Public teaching institution	4
Private teaching institution	2
Ministry of Health public hospital/clinic	4
Private hospital/clinic	2
*Total*	12

### Theme 1: perceived benefits of AI

This theme encompasses the positive perceptions and advantages associated with the use of AI in dental technology. The subthemes identified under this theme further elaborate on the specific benefits perceived by dental technicians.

### Subtheme 1: enhanced efficiency and productivity

Five dental technicians recognised that AI has the potential to enhance efficiency and productivity in dental laboratories. The automation of certain tasks, such as digital impressions and prosthesis design, was seen as a significant advantage. AI systems can perform these tasks faster and more accurately than traditional manual techniques, thereby saving time and increasing productivity.

P1: *“I think that AI can help us in produce dentures faster and better.”*

P3: *“We use CAD/CAM in our dental lab, and it does improve our productivity. We can save time and energy to fabricate more crowns and bridges.”*

P8: *“Of course, dentures and other lab works can be done faster with latest technologies.”*

P11: *“Digital scanner to take impression is way faster, we do not need to pour the dental cast, everything is just in the computer system.”*

P12: *“I believe with AI; it can help us increase our work productivity as they (AI technologies) are fast.”*

### Subtheme 2: improved accuracy and precision

Five Participants acknowledged that AI could contribute to improved accuracy and precision in the fabrication processes. AI algorithms can analyse data and patterns with a high level of precision, leading to more accurate diagnoses and treatment planning. The ability of AI to assist in CAD/CAM systems was specifically highlighted, as it can result in more precise and well-fitting dental restorations.

P2: *“CAD/CAM can produce better crowns and bridges, because we (dental technicians) are old, and we could not see the margin clearly.”*

P3: *“The intraoral scanner actually produces more accurate margins than the alginate impressions that doctors used to take.”*

P5: *“If there are more advanced machines, I think we can give better treatment for the patients.”*

P10: *“Our dental lab has CAD/CAM now, and we received good feedback from the dentists who sent their cases to our lab. They said our lab works become nicer, accurate.”*

P11: *“…with AI, our dentures will become better, more accurate, and patients will like our lab works.”*

### Subtheme 3: streamlined workflow and communication

AI was perceived as a tool that can streamline workflow and enhance communication between dental technicians and other members of the dental team. AI systems can facilitate the sharing of digital data, enabling seamless collaboration and reducing the need for physical transfers of information. This streamlined workflow was seen as beneficial in improving overall efficiency and ensuring effective communication among team members.

P2: *“I agree that with AI and advanced technology, our workflow will be better as things will be more systematic.”*

P4: *“With AI, we can share information with dentists even if our dental lab is far from the dental clinics.”*

P5: *“I find that AI might help a lot especially during times like the COVID-19 pandemic. We (dental technicians) do not need to communicate face-to-face with dentists. Everything is just through computer system.”*

P8: *“Ever since we have CAD/CAM in our clinic, our communication with dentists improved.”*

### Theme 2: concerns and challenges

This theme encompasses the concerns and challenges expressed by dental technicians regarding the use of AI in dental technology. The subthemes identified under this theme shed light on the specific areas of concern and potential challenges associated with the integration of AI.

### Subtheme 1: job security and professional identity

Six dental technicians expressed concerns about the impact of AI on job security and the future of dental technology as a profession. There was apprehension that the automation of certain tasks by AI systems might reduce the demand for manual craftsmanship skills traditionally associated with dental technicians. Participants raised questions about the evolving role of dental technicians in a technologically advanced landscape and the need to adapt their skills to remain relevant.

P2: *“I am afraid that dental technicians will be jobless in the future.”*

P3: *“If AI take over everything, does the world still need dental technicians?”*

P5: *“Of course our jobs will be at stake!”*

P7: *“In future, everything will be more advanced with AI and the demand for dental technicians will be reduced.”*

P9: *“I am wondering how we can adapt ourselves with the new technologies. How can we improve ourselves to remain in the market?”*

P11: *“There will not be any manual production of dentures… we will be replaced.”*

### Subtheme 2: ethical considerations

Five participants discussed ethical considerations related to the use of AI in dental technology. Privacy and data security emerged as significant concerns, as AI systems often require access to sensitive patient information. The ethical implications of data usage, storage, and sharing were highlighted, with an emphasis on the need for proper safeguards to protect patient confidentiality and comply with legal and regulatory requirements.

P1: *“Those high technological machines are good, but they might end up disclosing patients’ data.”*

P4: *“I do not trust AI because even my social media was easily hacked by others… So, how can we ensure that AI can protect our clients?”*

P5: *“One bad thing is that we can share patient cases easily and the case confidentiality cannot be adequately protected.”*

P6: *“I feel that no matter how good AI is, we still need some forms of regulation to safeguard patients’ information. We cannot sorely rely on AI.”*

P12: *“Even now, we sometimes share dental cases via telegram or WhatsApp, which is not totally ethical. Therefore, in the future it will be worst with advanced AI.”*

### Subtheme 3: training and support needs

Seven dental technicians stressed the importance of adequate training and ongoing support in using AI systems effectively. Participants highlighted the need for educational programs and professional development opportunities that address the specific skills required to work with AI in dental laboratories. The complexity of AI systems and the continuous evolution of technology necessitate continuous learning and support to ensure optimal utilisation and integration of AI in dental practice.

P4: *“If we want to secure our jobs in future, we need to have more training on how to use these technologies.”*

P7: *“The dental council should provide us with more training and support, allowing us to gain the skills in using AI in the lab.”*

P8: *“I hope to have more training in the future how to use these advanced technologies.”*

P9: *“There should be some postgraduate courses for dental technicians to guide us how to use latest technology.”*

P10: *“We need to have the proper professional development training.”*

P11: *“If we want AI to be incorporated into our dental technology systems, then the government need to send us for training.”*

P12: *“I wish to attend courses to increase my knowledge in the new machines so that I will be able to use it in the future.”*

## Discussion

The present study explored the perceptions of dental technicians regarding the use of AI in the field of dental technology. The results revealed two key themes which provide valuable insights into the potential advantages and obstacles associated with the integration of AI in dental laboratory practices. Participants acknowledged that AI has the potential to automate certain tasks, such as digital impressions and prosthesis design, leading to faster and more accurate results. This automation can save time and increase productivity in dental laboratories [7]. It has been found that AI improved the technique in prosthetic fabrication accuracy, such as the use of advanced 3D printing to print a variety of materials simultaneously with favourable detail reproducibility [21]. Participants also recognised that AI, like CAD/CAM systems could contribute to improved accuracy and precision in the fabrication processes. This observation aligns with outcomes from a prior study among dental technicians in the UK and Ireland [1]. AI algorithms can analyse data and patterns with a high level of precision, leading to more accurate diagnoses and treatment planning [22].

The integration of AI in the software allows the technicians to evaluate, design and fabricate the prosthesis by incorporating data from many actual prosthesis cases [7]. With prosthetic AI, more predictive measurements and precise outcomes can be witnessed, such as the re-establishment of the optimal maxillo-mandibular relationship in denture cases [23], generation of prosthetic crown morphology that is similar to the natural tooth [9], shades selection with precise colour matching to the adjacent natural teeth in the aesthetic zones [24, 25], and prediction of debonding possibilities of the CAD/CAM restorations [26]. Another advancement of AI can be seen in the fabrication of implant prostheses, whereby intraoral scans can be done by clinicians and inputted into the CAD/CAM software [27]. From there, the lab technicians can manufacture the surgical guide, determine the size of the implant screws and specification of the abutment up to the implant placements and the possible complications – all of which are understood by the CAD/CAM software [10]. Therefore, it is not surprising that most dental technicians in the current study perceived that AI can enhance the accuracy and precision of dental prostheses which further improve the quality and longevity of dental restorations [12], thereby benefiting patients and dental professionals alike.

In addition, participants perceived AI as a tool that can streamline workflow and enhance communication between dental technicians and other members of the dental team. The ability to expedite these processes allows dental technicians to focus their time and efforts on other critical tasks, ultimately improving overall efficiency in dental laboratories. AI systems can facilitate the sharing of digital data, enabling seamless collaboration and reducing the need for physical transfers of information [28]. This streamlined workflow was seen as beneficial in improving overall efficiency and ensuring effective communication among team members [29]. For instance, dental technicians can use AI-powered software to upload and share digital impressions, 3D scans, or CAD designs with dentists, prosthodontists, or other team members [30]. By streamlining communication and data exchange, AI can enhance interdisciplinary collaboration and improve the coordination of dental treatment [31].

While participants recognised the potential benefits of AI, they also expressed concerns about its impact on job security and the future of dental technology as a profession. There was apprehension that the automation of certain tasks by AI systems might reduce the demand for manual craftsmanship skills traditionally associated with dental technicians [12]. For instance, AI has the potential to substitute traditional methods like lost wax and casting techniques used by lab technicians with chairside intraoral scanning performed by dentists [32]. Additionally, in the milling process, AI-generated abutments or prosthetic frameworks necessitate fewer manual adjustments compared to conventional fabrication procedures [33]. The reduced influence of technicians could possibly be due to the overlapping of AI with the dental technicians’ job scope [7]. The present finding echoes concerns expressed in a previous published work regarding the potential displacement of human labour by AI in various industries [34]. Nonetheless, such a result contradicts the survey among UK and Irish technicians who did not worry that their role would be replaced by AI [1]. Despite many assumptions being made concerning AI that might replace human labour [35], there is still no evidence showing AI can completely replace the role of dental technicians.

There have also been potential ethical and technical challenges arising from AI applications in dentistry [36]. Ethical considerations of AI include patients’ informed consent and privacy, confidentiality, data protection, anonymity, and discrimination [37]. In the present study, participants highlighted ethical considerations related to the use of AI in dental technology, with a specific focus on privacy and data security. Participants also emphasised the importance of proper safeguards to protect patient confidentiality and comply with legal and regulatory requirements. The advancement of digital communication and social media might give rise to the falsification of data and misleading advertising [38]. Addressing these ethical considerations is crucial for the successful integration of AI in dental technology and ensuring patient trust in the use of AI systems. Nevertheless, AI’s decision-making capabilities can be questionable as the AI-based systems are run by computer scientists without proper healthcare backgrounds [39].

Dental technicians in the present study stressed the importance of adequate training and ongoing support in effectively using AI systems. Participants highlighted the need for educational programs and professional development opportunities that address the specific skills required to work with AI in dental laboratories. The complexity of AI systems and the continuous evolution of technology necessitate continuous learning and support to ensure optimal utilisation and integration of AI [40]. Organisations can offer workshops and seminars to provide hands-on training and demonstrations of new technologies specific to dental laboratories [41]. Technicians can learn directly from experts in the field and gain practical experience in using AI tools. Moreover, professional organisations and institutions may offer certification programs that validate the skills and knowledge of dental technicians in using latest technologies and machines [42]. Encouraging dental technicians to attend conferences and exhibitions focused on dental technology and AI is also another way to provide dental technicians with opportunities to learn about the latest advancements in the field. It is worth noting that providing comprehensive training programs and ongoing support can empower dental technicians to leverage the full potential of AI and overcome any barriers or challenges associated with its implementation.

The current study has certain limitations. First, the study included twelve dental technicians, which may not be representative of the entire population of dental technicians. A larger sample size could provide more diverse perspectives and increase the generalisability of the findings. Second, the participants were recruited from different dental laboratories within a single nation. Furthermore, the present study relied on self-reported data collected through interviews. The participants’ perceptions and perspectives on AI in dental technology may be influenced by personal biases, experiences, and expectations. The participants who were included in the study obtained a basic dental technology diploma, and future research can include dental technicians who have bachelor’s degrees since they are exposed to more sophisticated dental equipment and technologies throughout their undergraduate bachelor’s course [2]. Dental technicians with bachelor’s degree were not included in the present study due to the historical context of dental technology education in Malaysia. There is only one private dental institute introduced a Bachelor of Dental Technology program in Malaysia [2], and the first batch of graduates from this program emerged in 2019. Given this timeline, the first cohort of locally trained dental technicians with bachelor’s degrees possesses relatively limited experience in the field, having accumulated less than five years of practical engagement. Hence, this consideration guided our decision to exclude this group from the current study.

Nevertheless, the findings of this qualitative study provide valuable insights into the perceptions of dental technicians regarding the use of AI in dental technology. By exploring their concerns, and considerations, this research sheds light on the potential benefits and challenges associated with the integration of AI in dental laboratory practices. In addition, the findings of this study have implications for dental technology education, dental laboratory management, and policy development. Education and training programs should incorporate AI-related knowledge and skills to prepare dental technicians for the evolving technological landscape [2]. Dental laboratories and professional associations should address concerns related to job security and ethical considerations through guidelines and policies. Collaboration between dental technicians, researchers, and AI developers can facilitate the development of AI systems tailored to the needs and requirements of dental laboratory practice. Further research can explore the perceptions and experiences of other stakeholders in dental practice, such as dentists, patients, and regulatory bodies. Additionally, quantitative studies can be conducted to assess the actual impact of AI implementation on efficiency, accuracy, and patient outcomes in dental laboratories.

## Conclusion

The perceptions of dental technicians regarding the use of AI in dental technology revealed both perceived benefits and concerns. Dental technicians acknowledged the potential of AI to enhance efficiency, accuracy, and communication in dental laboratories. However, concerns related to job security, ethical considerations, and the need for training and support were also raised. The successful implementation of AI in dental technology requires addressing these concerns and challenges through comprehensive education and training, ethical guidelines, and interdisciplinary collaboration. By understanding the perspectives of dental technicians, dental professionals can effectively integrate AI into dental laboratory practices, leading to improved patient care and outcomes in the field of dental technology.

## Data Availability

All data generated or analysed during this study are included in this published article.

## Abbreviations

AI: Artificial Intelligence
CAD/CAM: Computer-aided Design / Computer-aided Manufacturing
COREQ: Consolidated Criteria for Reporting Qualitative Research
TAM: Technology Acceptance Model
AIMST: Asian Institute of Medicine, Science and Technology University
